# Increasing vitamin C through agronomic biofortification of arugula microgreens

**DOI:** 10.1038/s41598-022-17030-4

**Published:** 2022-07-30

**Authors:** Shivani Kathi, Haydee Laza, Sukhbir Singh, Leslie Thompson, Wei Li, Catherine Simpson

**Affiliations:** 1 Department of Plant and Soil Science, Texas Tech University, Lubbock, TX 79409 USA; 2 Department of Animal and Food Sciences, Texas Tech University, Lubbock, TX 79409 USA; 3 Department of Chemical Engineering, Texas Tech University, Lubbock, TX 79409 USA

**Keywords:** Nutrition, Plant physiology, Abiotic

## Abstract

Vitamin C (Vit C) is an essential micronutrient and antioxidant for human health. Unfortunately, Vit C cannot be produced in humans and is ingested through diet while severe deficiencies can lead to scurvy. However, consumption is often inconsistent, and foods vary in Vit C concentrations. Biofortification, the practice of increasing micronutrient or mineral concentrations, can improve the nutritional quality of crops and allow for more consistent dietary levels of these nutrients. Of the three leading biofortification practices (i.e., conventional, transgenic, and agronomical), the least explored approach to increase Vit C in microgreens is agronomically, especially through the supplemental application of ascorbic acid. In this study, biofortification of Vit C in microgreens through supplemental ascorbic acid was attempted and proven achievable. Arugula (*Eruca sativa 'Astro'*) microgreens were irrigated with four concentrations of ascorbic acid and a control. Total Vit C (T-AsA) and ascorbic acid increased in microgreens as supplementary concentrations increased. In conclusion, biofortification of Vit C in microgreens through supplemental ascorbic acid is achievable, and consumption of these bio-fortified microgreens could help fulfill the daily Vit C requirements for humans, thereby reducing the need for supplemental vitamins.

## Introduction

Human diets require supplementation of various nutrients to prevent the onset of various diseases and disorders. One of the most common diseases associated with nutrient deficiencies is scurvy, caused by Vitamin C (Vit C) deficiency, and leads to symptoms such as detached gums, bleeding through nostrils, bleeding gums, ulcerations on legs, anemia, fatigue and depression, which if left untreated can be fatal^1^. Though Vit C deficiencies and scurvy are rarely seen in developed countries such as the USA, the reported prevalence of Vit C deficiencies is between 1.4 and 20% in European nations and between 3 and 16% in North America^2^. In the primary populations suffering deficiencies are the elderly and those in low and middle income countries where the prevalence of deficiencies increases to up to 78%^2^. However, Vit C deficiencies are difficult to diagnose as they initially present with non-specific symptoms such as depression and anxiety^3^. Meeting the dietary needs of different populations can be challenging due to poverty or accessibility. Research on increasing the intake of Vit C through agronomic crops has expanded for many reasons. One of the most important reasons being that Vit C is not biosynthesized by humans, and so must be consumed^4^. Ever since the discovery that Vit C plays major role in several enzymatic reactions that involve collagen hydroxylation and biosynthesis of hormones and neurotransmitters, the recommended dietary intake for adults was increased from 10 mg^5^ to 75 and 90 mg day^-1^ for adult women and men in the US, but also varies by life stage, gender, and pregnancy^6,7^. The main sources of Vit C for humans are through plants or plant-based foods such as citrus fruits, camu-camu berries, broccoli, kiwi, pepper, tomatoes^8^. In vegetables, Vit C is naturally present in the form of ascorbic acid (AsA) and its oxidized form, dehydroascorbic acid (DHA). The sum of AsA and DHA constitutes total Vit C because DHA can be taken up by the facilitative glucose transporters in the small intestine and interchanged to AsA under favorable conditions^9–12^.

According to the United Nations Department of Economic and Social Affairs^13^, the world’s population is estimated to reach 9.1 billion by 2050, which requires an increased food production by approximately 70% to achieve food security. However, climate change, urban encroachment and the declining arable land due to reduced fertility and degradation is resulting in production of lower quality foods that are not as nutrient dense^14^. Furthermore, ‘hidden hunger’ or ‘hidden undernutrition’ due to the lack of essential micronutrients and resulting dietary quality have caused widespread problems^15,16^. This calls for the need to enhance the nutritional quality of plants and plant products. Biofortification is a sustainable approach of improving the nutritional quality of plant biomass thereby increasing their bioavailability to humans and has potential to offer long-term solutions to minimize food insecurity and malnutrition^17^. However, limited data are available on biofortification of vitamins in agronomic production^18,19^. In plant production and research, ascorbic acid has been used in growing media as a practice to improve plant parameters such as growth and shoot length^20^ and as a foliar spray to lower absorption of sodium (Na), reduce salinity stress^21^, other environmental stressors, and to protect plants from damage caused by air pollution^22,23^. Foliar application of ascorbate salts has also increased ascorbic acid in plants such as beans, lettuce, petunia and tomato when measured 24–72 h after application^22,24–26^. However this increase in ascorbic acid in plants disappeared by the time of harvest suggesting that ascorbic acid metabolism could be closely tied to the carbohydrate metabolic pool and is used quickly in cellular processes^23^. Other evidence has shown that while some short-term enhancement is seen, Vit C fertilization has been ineffective beyond early growth stages, particularly in mature plants^20,27^. Due to these non-persistent results, agronomic biofortification of Vit C through supplemental ascorbic acid has not been widely pursued, although biofortification through genetic engineering has been explored to a larger extent^18,19,23,28,29^, However, this process is time consuming and cost prohibitive to many of the target populations in need of nutritionally dense crops. These previous findings led us to question whether short-term vegetative crops can be biofortified to a greater extent compared to crops whose edible parts are consumed at maturity. Exploring the ability of plants to absorb Vit C during early growth stages and harvesting them before degradation could be an answer to providing a more stable supply of Vit C to humans. Microgreens are an emerging crop that is consumed at very early growth stages, and have gained in popularity in recent years^30^. Microgreens are known for their nutrient density and can be harvested between 7 and14 days upon full development of cotyledonary leaves. Some microgreens, like roselle, basil, fenugreek, can provide up to 116% of the reference daily intake of vitamins like C, E, and beta-carotene^31^. Of the few published studies on biofortification of microgreens, researchers found that biofortification of Zn, Fe, Se and I were possible. For example, Di Gioia et al.^32^ found success in biofortifying microgreens with Zn and Fe supplemental solutions, but with species specific responses. Similarly, Puccinelli et al.^33^, and Newman et al.^34^, have successfully increased Se in herbs grown as microgreens. Germ et al.^35^, also had success in increasing Se and I in common buckwheat microgreens. Yet, the biofortification of other nutrients such as Vit C remain unstable and fluctuate relative to environmental conditions^36^. Most of the current literature concludes that bioengineering and breeding are the most promising ways to increase Vit C content in agronomic crops^18,19,23,28,29^.

Yet, it is imperative to think of practical solutions to not only improve nutrition, but access to nutrient dense crops to the growing population. Arugula (*Eruca sativa*), a specialty cool-season Brassicaceae crop known for its spicy-pungent leaves, was chosen for this study due to its availability and widespread use^37^. Arugula microgreens are typically consumed in salads, as garnishes, or additions to other food products for added flavor and aesthetic appeal. Total ascorbic acid concentration of arugula has been recorded as approximately 45.8 ± 3.0 mg 100 g^−1^ fresh weight (FW)^38^. Improving this to levels equivalent to or exceeding what is found in citrus or peppers (50–80 mg 100 g^−1^ FW) would provide an accessible, Vit C rich option for deficient populations. Therefore, the objectives of this study were to determine if Vit C content could be increased in a short-term leafy green crop such as arugula through ascorbic acid supplementation and assess if any increases persist to the stage of consumption.

## Results

### Effect of ascorbic acid enrichment on Vit C in microgreens

**Figure 1:**
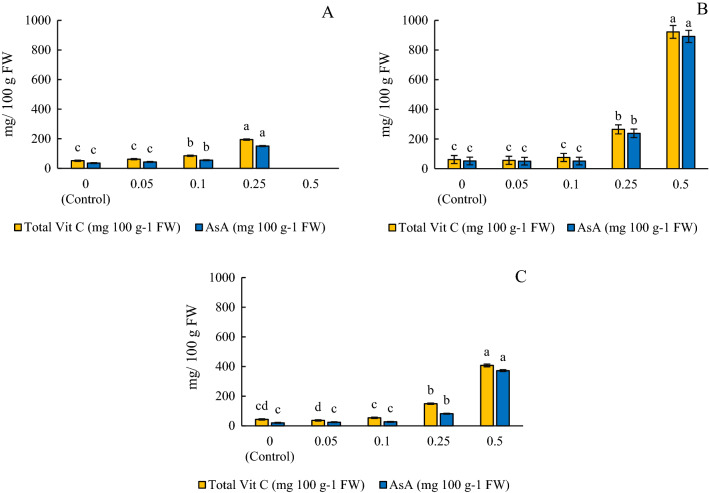
Average of concentrations of total Vit C and ascorbic acid (AsA) in fresh weight (FW) of each treatment treated with different concentrations of ascorbic acid in (**A**) Experiment 1—greenhouse conditions, (**B**) Experiment 2—greenhouse conditions, and (**C**) Experiment 3—laboratory conditions. Significant differences (*p* ≤ 0.05) among treatments are indicated by different lowercase letters within the experiment. (n = 5). Bars represent ± 1 standard error of the mean. *FW* fresh weight.

The total Vit C and AsA concentrations in the microgreens were significantly different across the three experiments (*p* < 0.0001). However, this could have been affected by missing data from the death of samples in the 0.5% treatment in experiment 1 or varying temperature and light conditions due to microgreens being grown in greenhouse versus lab conditions. The overall results in each experiment show that, with the increase in the applied concentration of ascorbic acid there was a proportional increase in the concentration of both total Vit C and AsA of the microgreens (Fig. 1; Tables 1 and 2). There was also a significant increase in Vit C in microgreens treated with versus without ascorbic acid in all the three experiments (*p* ≤ 0.0001). Treated microgreens had higher concentrations of Vit C with the exception of 0.05% and 0.1% treatments in experiment 2 and 3, which were not significantly different from the control. The highest concentrations of total Vit C and AsA per 100 g FW were seen in the microgreens treated with the highest concentrations of ascorbic acid (407–922 mg 100 g^−1^ FW and 372–891 mg 100 g^−1^ FW, respectively) and the lowest concentrations were in the control (0%) and 0.05% ascorbic acid treatments (36–61 mg total Vit C 100 g^−1^ FW and 20–51 mg AsA 100 g^−1^ FW, respectively) (Fig. 1). Ultimately, the application of various rates of ascorbic acid resulted in increases of between 20 and 840% total Vit C, and 20–1727% AsA in arugula microgreens (Table 1).

**Table 1:** Average percent change of total Vit C and ascorbic acid (AsA) in each treatment treated with different concentrations of ascorbic acid over 3 different experiments.

Treatment (% ascorbic acid)	Experiment-1 (greenhouse)		Experiment-2 (greenhouse)		Experiment-3 (lab)	
	% Increase total vit C	% Increase AsA	% Increase total vit C	% Increase AsA	% Increase total vit C	% Increase AsA
0 (Control)	–	–	–	–	–	–
0.05	+ 20.0	+ 22.0	− 8.6	− 2.3	− 15.0	+ 20.1
0.1	+ 65.2	+ 55.2	+ 23.0	− 1.5	+ 24.5	+ 33.0
0.25	+ 277.0	+ 324.1	+ 331.2	+ 360.6	+ 244.9	+ 302.1
0.5	–	–	+ 1402.3	+ 1625.9	+ 840.5	+ 1727.5

Significant differences (*p* ≤ 0.05) among treatments are indicated by different lowercase letters within the experiment. (n = 5).

*FW *fresh weight.

**Table 2:** Fresh weight and dry weight of microgreens treated with application of different concentrations of ascorbic acid in 3 experiments.

Treatment (% ascorbic acid)	Experiment-1	Experiment-2		%DM	Experiment-3		%DM
	Fresh weight (g)	Fresh weight (g)	Dry weight (g)		Fresh weight (g)	Dry weight (g)	
0 (Control)	4.60	7.44ab	0.91a	12.2	8.62 a	0.81	9.4
0.05	5.23	7.54ab	0.91a	12.1	7.53 a	0.84	11.1
0.1	5.31	8.08a	1.02a	12.6	8.89 a	0.90	10.1
0.25	5.43	2.62b	0.84ab	32.1	7.92 a	0.92	11.6
0.5	–	0.66b	0.61b	92.4	3.82 b	0.96	25.1
*P-*value	0.9369	0.0001*	0.0028*	–	0.0031*	0.0874	

Significant values are in italics.

*DM *dry matter.

*Significant differences (*p* ≤ 0.05) among treatments are indicated by different lowercase letters within the column.

### Effect of ascorbic acid enrichment on biomass

The fresh weight (FW) yields of arugula microgreens differed significantly between the three experiments (*p* = 0.009), but the dry weight (DW) did not differ significantly in the second and third experiments (*p* = 0.645). Dry weight was not recorded for experiment 1 as it was an exploratory study. In experiments 1 there were no significant effects of treatment on FW. In experiment 2 and 3, the FW differed significantly between the treatments, with the highest in the 0.1% treatment and the lowest in the 0.5% treatment. While larger concentrations of Vit C and AsA were found in microgreens treated with 0.5% ascorbic acid, these plants saw significant, negative impacts on plant health (i.e., wilted plants) and biomass (Table 2). This indicates that harvested yields decline at 0.5% ascorbic acid application rates; at lower rates yields were not impacted or were increased.

### Effect of ascorbic acid enrichment on chlorophylls and carotenoids

Chlorophyll and carotenoid concentrations were significantly different in each experiment (Table 3). However, in all experiments, carotenoids were not significantly affected by ascorbic acid treatments. In experiment 1, there was no significant effect of treatment on chlorophylls a or b. However, in experiments 2 and 3, chlorophylls a and b were significantly affected by treatment concentrations. The lowest chlorophyll concentrations were consistently in the 0.5% ascorbic acid treatment. In the lower application concentrations of ascorbic acid, differences between chlorophylls a and b were minimal. The highest chlorophylls were found in the 0.1% ascorbic acid treatment for experiment 2. In experiment 3, the highest chlorophyll a concentrations were found in the 0.1% ascorbic acid treatment while the highest chlorophyll b concentrations was in the control treatment. Thus, biofortification using ascorbic acid increased chlorophylls, which has positive impacts on plant performance and nutrition^39^.

**Table 3:** Chlorophyll-a, Chlorophyll-b and carotenoids in microgreens treated with different application concentrations of ascorbic acid in 3 experiments.

Treatment (% ascorbic acid)	Experiment-1			Experiment-2			Experiment-3		
	Chl-a	Chl-b	Carotenoids	Chl-a	Chl-b	Carotenoids	Chl-a	Chl-b	Carotenoids
0 (Control)	13.23	6.72	3.09	20.28a	10.38ab	4.48	25.56a	20.37a	3.74
0.05	13.35	6.41	3.33	18.25ab	10.15ab	3.93	23.18a	19.81bc	5.14
0.1	12.91	5.38	3.54	24.57a	15.78a	4.66	25.95a	16.29a	4.19
0.25	15.04	6.33	3.92	11.37bc	4.38b	3.52	24.95a	12.98ab	4.92
0.5	–	–	–	9.117c	3.67b	2.85	13.30b	5.85c	3.79
*P*-value	0.889	0.864	0.501	< *0.0001*	*0.0017*	0.0571	< *0.0001*	*0.0258*	0.2967

Significant values are in italics.

Significant differences (*p* ≤ 0.05) between treatments are indicated by different lowercase letters within the column.

### Effect of ascorbic acid enrichment on K concentration in microgreens

**Figure 2:**
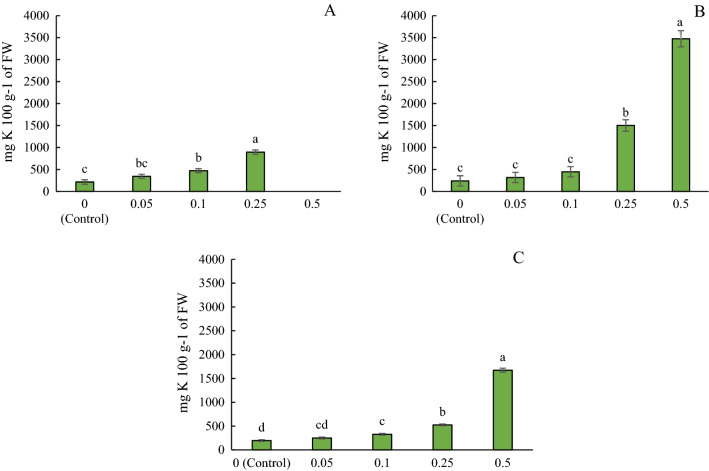
Potassium concentrations in microgreens treated with ascorbic acid and KOH buffer in (**A**) Experiment 1—greenhouse conditions, (**B**) Experiment 2—greenhouse conditions, and (**C**) Experiment 3—laboratory conditions. Significant differences (*p* ≤ 0.05) among treatments are indicated by different lowercase letters within the experiment. (n = 5). Bars represent ± 1 standard error of the mean. *FW* fresh weight.

To buffer the solutions to a non-lethal pH, KOH was used to increase the pH of nutrient solutions with ascorbic acid to an approximate pH of 6. The amount of KOH needed to increase the pH increased with increasing concentrations of ascorbic acid in the treatments. It was suspected that increased levels of K in the nutrient solution may have resulted in increased K concentrations in the microgreens. To verify this, the K concentration of the microgreens was analyzed. As suspected, the results showed significantly increased levels of K in microgreens treated with higher concentrations of ascorbic acid and KOH (Fig. 2). The highest K was found in 0.5% ascorbic acid (i.e., highest concentration of ascorbic acid applied). There were significant differences in K among the different experiments (*p* < 0.0001). Increased K can be beneficial for plants and humans as K improves stomatal functioning and water regulation in plants^40^ and reduces blood pressure in humans^41^. However, increased K is also necessary to consider in people that require low K diets^42^.

### Differences in EDI and NC of Vit C and K in all the treatments

EDI and NC are significant to human nutrition because they indicate daily requirements of vitamins and nutrients, as well as nutritional quality of plants. These are important to consider when biofortifying plants to ascertain how they relate to human health. Thus, we included these calculations to put our findings into perspective. Estimated daily intake and NC of both Vit C and K increased with the increase in the concentration of ascorbic acid in the treatments. The highest EDI for Vit C and K was seen in the 0.5% ascorbic acid treatment (i.e., 565.10 and 2184.5 mg day^−1^ respectively) whereas the lowest values were seen in 0.05% for Vit C (43.87 mg day^−1^) and control for K (184.17 mg day^−1^). The highest NC for Vit C and K was seen in the 0.5% ascorbic acid treatment (i.e., 627.89 and 46.48%) whereas the lowest was seen in 0.05% for Vit C (48.74%) and control for K (3.92%) (Table 4). At an application rate of 0.25%, EDI was 172.38 mg day^−1^ which translates to 191% NC; meaning that consuming less than FDA recommended RACC would meet daily Vit C dietary needs. And, consuming Vit C biofortified microgreens can help individuals achieve this daily dietary requirement of Vit C with considerably lower quantities of microgreens.

**Table 4:** Average of estimated daily intake and nutrient contribution of Vit C and K for all the treatments.

Treatments (% ascorbic acid)	EDI of vit C (mg day^−1^)	EDI of K (mg day^−1^)	NC of vit C (%)	NC of K (%)
0 (Control)	44.27	184.17	49.19	3.92
0.05	43.87	257.83	48.74	5.49
0.1	60.79	354.17	67.54	7.54
0.25	172.38	824.50	191.53	17.54
0.5	565.10	2184.50	627.89	46.48

Because these calculations are functions of presented data, no statistical analysis was conducted.

*EDI* estimated daily intake, *NC* nutrient contribution.

## Discussion

**Figure 3:**
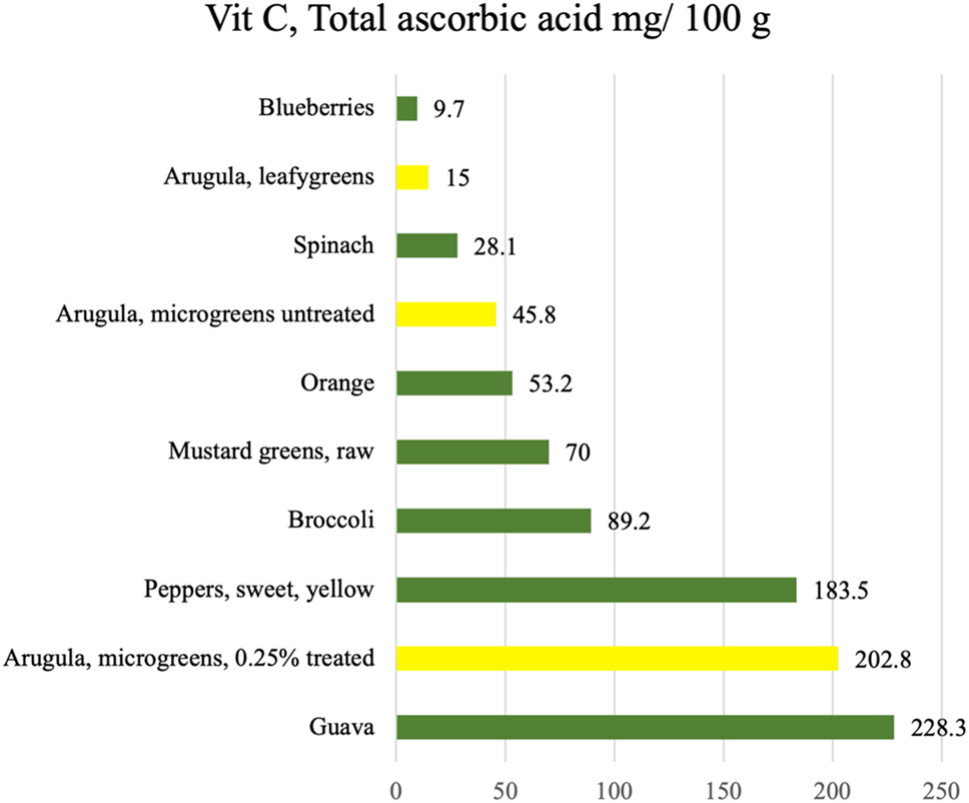
Vit C, Total ascorbic acid content (mg) 100 g^−1^ of a range of horticulture crop plants consumed raw.Reported content is that found in the raw and edible organs of the plants and was adapted from the United States Department of Agriculture database^53^ (ndb.nal.usda.gov), with the exception of Arugula, microgreens, untreated^38^ and Arugula, microgreens, 0.25% treated.

The purpose of our research was to determine if microgreens can take up ascorbic acid from nutrient solutions and have measurable accumulation at its stage of consumption. The results showed that biofortification of Vit C in plants at early growth stages is possible, and the concentrations of Vit C increased with an increase in the ascorbic acid concentration applied. Our preliminary studies determined that over 0.25% ascorbic acid was the threshold for survival in arugula microgreens, at and above which mortality increased dramatically. At lower rates, the increase in Vit C was stable, consistent, and retained long enough to be seen post-harvest. Microgreens are consumed at early plant developmental stages, making this a more relevant and feasible method for increasing production and consumption of biofortified plants. Previous studies have shown that arugula microgreens have concentrations of total ascorbic acid at approximately 45.8 mg 100 g^−1^ FW^38^. Comparatively, the highest concentrations of Vit C (264.68 mg 100 g^−1^ FW) observed in these experiments (without affecting biomass) was, almost six-fold greater than what has been reported in previous studies. For reference, oranges contain approximately 50 mg Vit C 100 g^−1^ FW (Fig. 3)^36^. In this study, concentrations of total Vit C varied from 36 to 922 mg 100 g^−1^ FW but increased as supplementary ascorbic acid concentrations increased. This resulted in an average 289.4% increase of total Vit C in microgreens at the 0.25% treatments compared to the control. Although the environmental conditions did affect the concentrations of microgreens across the three experiments, it is important to note that the patterns remained the same i.e., Vit C concentration in microgreens increased with the increased rate of application of ascorbic acid in the nutrient solution.

The average total Vit C in 0.5% treatments was 664.82 mg 100 g^−1^ FW whereas the average total Vit C in the control was 52.08 mg 100 g^−1^ FW. Although the 0.5% ascorbic acid treatment had the greatest amount of Vit C, the microgreens were almost completely wilted and had less fresh biomass which likely concentrated the samples. The mortality of microgreens at 0.5% was likely due to a higher ascorbic acid concentration in the solution which resulted in increased EC from the addition of more solutes in the solution. Therefore, the threshold concentrations for ascorbic acid supplementation lie between 0.25 and 0.5%; above which may be detrimental to plant growth. Thus, 0.25% is the recommended concentration for optimal biofortification of arugula microgreens. At application rates of 0.25% ascorbic acid, total Vit C and AsA concentrations were 149–264 mg 100 g^−1^ FW and 81–264 mg 100 g^−1^ FW, respectively, which equates to an average increase of 289.4% and 337.0% compared to the control (43–61 mg 100 g^−1^ FW and 20–51 mg 100 g FW^−1^, respectively) (Fig. 1 and Table 1). Improvement of total Vit C and ascorbic acid concentrations can also be achieved at lower application rates.

While 0.5% ascorbic acid treatments resulted in higher EDI and other factors, the increased mortality does not make them a feasible candidate for consumption, thus, we will discuss further results based on the 0.25% application rate as optimal. The EDI for Vit C was the highest in 0.25% ascorbic acid treatment with 172.38 mg day^−1^ and was the lowest in the control and 0.05% ascorbic acid treated microgreens with 44.27 and 43.87 mg day^−1^, respectively, meaning that consumption of 100 g of 0.25% ascorbic acid treated microgreens provide 172.38 mg of Vit C per day whereas 100 g of untreated microgreens provide 44.27 mg of Vit C per day. The percent nutrient contribution for Vit C was the greatest in 0.25% ascorbic acid treatments with 191.53% NC and the lowest in 0.05% ascorbic acid and control treatments with 48.74% and 49.19% NC, respectively. Furthermore, we observed significant increases in K as the concentrations of K in the buffer solution increased. The EDI and NC of K was greatest in the 0.25% ascorbic acid treatment with 2184.5 mg day^−1^ and 46.5% NC, and lowest in the control with 184.2 mg day^−1^ and 3.92% NC, respectively. While K was not the focus of this study, it was used to buffer the ascorbic acid solution to a pH more suitable for plant growth. As the concentration of ascorbic acid increased, more KOH buffer was needed to balance the pH. KOH was chosen as a buffer because sodium hydroxide may have induced toxic results and osmotic stress. While increased K can be beneficial or detrimental depending on the presence of certain chronic diseases^43^, this demonstrates that other essential nutrients can be taken up in conjunction with ascorbic acid. But, K is known to be taken up in excess by plants due to its use in water regulation, so this may not be the case with all mineral nutrients^44^. However, the increased uptake of K could also be due to the presence of ascorbic acid as it is known to increase uptake of several micronutrients such as Fe, Zn, and Ca by the plants^45^. Further research is needed to determine if KOH has any role in the increase of Vit C and if the presence of ascorbic acid in nutrient solutions had any effect on uptake of other nutrients. Due to limited sample volume, this could not be conducted in this study.

Overall, these experiments show that microgreens can be biofortified with ascorbic acid and K to increase concentrations within plant tissues and improve nutritional quality of plants. Only a few studies have used ascorbic acid to increase Vit C in plants in younger stages of the crop^27^, but the effects do not last longer than 72 h after ascorbic acid application^22,24–26^. Our research conflicts with these previous studies in that the Vit C concentrations were increased in the crop at the stage at which it would be consumed. Furthermore, the concentrations indicate that cumulative absorption is occurring as evidenced by the high concentrations found in tissues. While the mechanisms are not fully known, we hypothesize that osmotic stress due to high EC (Table 5) or the greater nutrient flux during plant early growth stages may cause increased absorption of these compounds^46,47^.

**Table 5:** Amount of water and ascorbic acid applied to microgreens in each treatment.

Experiment	Treatment (% ascorbic acid)	EC of the solution (µS cm^−2^)	Amount of treatment applied (mL) day^−1^ at each application														Total treatment volume applied (mL)	Total ascorbic acid applied (mg)	Total K applied (mg)
			1	2	3	4	5	6	7	8	9	10	11	12	13	14			
1	0	417	50	50	–	25	50	–	30	H	–						205	0	13.3
	0.05	674	50	50	–	25	50	–	30								205	102.5	38.9
	0.1	920	50	50	–	25	50	–	30								205	205.0	72.6
	0.25	1610	50	50	–	25	50	–	30								205	512.5	102.5
	0.5	2800	50	50	–	25	50	–	30								205	1025.0	235.7
2	0	417	30	–	30	–	40	–	30	–	30	30	30	20	H	–	240	0	15.6
	0.05	652	30	–	30	–	40	–	30	–	30	30	30	20			240	120.0	46.6
	0.1	920	30	–	30	–	40	–	30	–	30	30	30	20			240	240.0	93.6
	0.25	1604	30	–	30	–	40	–	30	–	30	30	30	20			240	600.0	120.0
	0.5	2800	30	–	30	–	40	–	30	–	10	20	30	20			210	1050.0	245.7
3	0	417	30	–	30	30	–	–	30	–	30	–	25	–	20	H	195	0	13.3
	0.05	684	30	–	30	30	–	–	30	–	30	–	25	–	20		195	97.5	35.9
	0.1	952	30	–	30	30	–	–	30	–	30	–	25	–	20		195	195.0	60.8
	0.25	1675	30	–	30	30	–	–	30	–	30	–	25	–	20		195	487.5	95.5
	0.5	2770	30	–	30	30	–	–	30	–	30	–	25	–	20		195	975.0	226.2

Treatments were started after the appearance of cotyledons and terminated at harvest.

Cells marked with ‘H’ indicate when microgreens were harvested.

While Vit C did not affect chlorophyll a, b and carotenoids in experiment 1, chlorophylls were significantly decreased by higher concentrations of ascorbic acid in experiments 2 and 3. This was likely due to wilting and death of microgreens observed at the highest treatment concentration. Ultimately, biofortification of arugula microgreens by enriched ascorbic acid nutrient solution is possible and can be easily achieved. This method results in more nutritious microgreens which can provide a higher percentage of the dietary requirement of Vit C in a smaller amount of consumed microgreens. This leads to wide ranging benefits from higher accessibility of more nutritious fresh foods, and the potential for biofortification of other essential vitamins and nutrients.

## Materials and methods

### Experimental setup

Three experiments were conducted to determine the feasibility and concentrations of ascorbic acid uptake into arugula microgreen tissues. The first two experiments were conducted in the Texas Tech University Horticultural Greenhouses (33.58407°N, 101.88691°W) in Lubbock, Texas. The last experiment was conducted in controlled laboratory conditions in the Bayer Plant Sciences Building (33.58213°N, 101.87890°W) at Texas Tech University. The first two experiments were conducted under shade cloth with the average temperatures being ~ 21 ºC and during the months of Nov-Dec and Feb 2021. The final experiment was conducted at room temperature (approximately 22 °C) with supplementary LED lighting with PAR ranging approximately 60–68 µmol m^−2^ s^−1^ each day. Different conditions were used to determine if environment affected Vit C concentrations in plants.

Commercially produced seeds of arugula (*Eruca sativa ‘Astro’*; Johnny’s Seeds, Fairfield, Maine, USA) were used for all three experiments. Seeds complied with relevant guidelines on the collection and permissions for seed specimens. Arugula seeds were sown at a rate of 1.5 g tray^-1^ onto a growing pad (Micromat, Salt Lake City, Utah, USA) in 12.7 × 12.7 cm^2^ plastic trays with lids. These seeds were misted with deionized water and covered with a clear lid to maintain humidity and facilitate germination. Five days after germination, the lids were opened, and treatments were applied. Five treatments were used differing in the ascorbic acid and KOH concentrations. The treatments include: Floragro solution (N–P–K–Mg = 2–1–6–0.5; General Hydroponics, Santa Rosa, California, USA) with 0.05% ascorbic acid (L-(+)-Ascorbic acid, Alfa Aesar, Haverhill, Massachusetts, USA), 0.1% ascorbic acid, 0.25% ascorbic acid, 0.5% ascorbic acid and 0% ascorbic acid (Control). The Floragro solution was mixed with the treatments of ascorbic acid at a concentration of 132 mL 100 L^−1^. Solutions were buffered with KOH to a pH of approximately 6 before application to germinated seeds. These five treatments were replicated five times in a completely randomized design and each container was considered to be a technical replicate. The growing pads were fertigated with treatment solutions to maintain adequate moisture based on pad saturation until harvest and volume was recorded at each irrigation event (Table 5).

### Plant measurements

Approximately 11–18 days after sowing (DAS), the microgreens had turgid cotyledons with the tips of the first true leaves had appeared. They were then harvested by cutting the shoots approximately 2 mm above the surface of growing pad using sterilized scissors. The fresh biomass of each treatment was weighed, freeze-dried, (HarvestRight, North Salt Lake City, Utah, USA) and weighed again. The dried biomass was then ground in the presence of liquid nitrogen and stored at−80 °C until further analysis.

### Chemical analyses

#### Vitamin C extraction and analysis

Vit C extraction from the freeze-dried samples was performed using 6% trichloroacetic acid as described by Sérino et al.^48^, who adapted Vit C analysis protocols for microplate spectroscopy. Following the extraction, 20 µL of each sample was added to the microplate and reacted using dithiothreitol (DTT) to reduce oxidized ascorbate. Samples were prepared simultaneously for both T-AsA and AsA and absorbance was read at 550 nm using the microplate spectrophotometer (SpectroMax iD3, San Jose, California). Total Vit C and AsA 100 g^−1^ FW were calculated according to Sérino et al.^48^.

#### Chlorophyll and carotenoid analysis

The determination of chlorophylls a, b, and carotenoids in the samples was performed according to Lichtenthaler^49^ with modifications for microplate spectroscopy. Briefly, 10 mg of freeze-dried and ground microgreens were measured and placed in 5 mL test tubes, chlorophyll a, b, and carotenoids were then extracted using 1 mL of 100% methanol. The samples were mixed for 3 min and 200 µL of the filtrate was placed in each microplate well. Methanol was used as a blank and the absorbance was recorded using the microplate spectrophotometer (SpectroMax iD3, San Jose, California). The absorbance was read at 665, 652, and 470 nm for chlorophyll a and b and carotenoids, respectively.

#### K analysis

To determine K concentrations, samples were first prepared by ashing in a muffle furnace at 550 °C overnight (12 h). The fully combusted samples were then digested using 3 mL of 80% nitric acid and 3 mL of DI water. The clear layer left after digestion was allowed to sit in 10 mL of 20% nitric acid overnight. The next day, the samples were filtered, and final volume was brought up to 50.0 mL using DI water. These samples were then analyzed using ICP-OES (iCAP 7600, Thermo Fisher, Waltham, Massachusetts, USA).

#### Estimated daily intake (EDI) and nutrient contribution (NC) analysis

Estimated daily intake is the maximum amount of nutrients that should be eaten on an average per day and NC is the nutrient contribution of the microgreens to each meal^31^. EDI and NC were calculated to determine their nutrient contribution (Eq. 1). Estimated daily intake was determined using nutrient composition results from this study against a FDA reference amount customarily consumed (RACC)^31^. As in previous studies^31,50^, RACC was used for their mature forms i.e., leafy greens (85 g) since the RACC for microgreens is yet to be determined^51^.

Estimated daily intake equation:

1 $$EDI \,\left( {\frac{mg}{{day}}} \right) = \frac{{Nutrient\; content \left( {mg/100g} \right)}}{100}*RACC \,\left(g \right)$$

Reference daily intake (RDI) for K and Vit C were then used to calculate the NC (Eq. 2; Ghoora et al.^31^). RDI of K and Vit C for adults is 4700 mg day^−1^ and 90 mg day^−1^ respectively^52^.

Nutrient contribution equation:

2 $$Nutrient\; contribution \,\left( \% \right) = \frac{EDI}{{RDI}}*100$$

### Statistical analysis

Statistical analysis was performed using JMP Pro 16.0.0 (SAS Institute, Cary, NC). Significant differences between treatments were determined at *P* ≤ 0.05 using factorial analysis and standard least squares regression models. Mean separation was determined using Tukey’s tests.

## Conclusion

Application of ascorbic acid as a part of a nutrient solution increases total Vit C and AsA in plants harvested at a young growth stage such as microgreens. While consumption and palatability are yet to be tested, we hypothesize that these Vit C biofortified microgreens could help reduce Vit C deficiency in humans if consumed in sufficient quantities. Daily dietary intake requirements of Vit C can be achieved by consuming a much lower volume of microgreens after biofortification. Furthermore, microgreens can be easily grown in individual homes, thereby making them more accessible and potentially consumed more frequently.

## Data Availability

Data is available upon request. To request data, please send an email to the corresponding author, Dr. Catherine Simpson.

## Material availability

All plant materials and procedures were conducted in accordance with institutional, national, and international guidelines and legislation.
